# In Memoriam

**Published:** 1889-06

**Authors:** 


### IN MEMORIAM—GEORGE F. FOOTE.

Many of our readers will hear with deep regret of the sudden death of Dr. George F. Foote, which occurred at Chicago, May 8th, 1889.

Dr. Foote has been practicing Homœopathy for many years, he being one of the earliest members of the American Institute. He was formerly a professor in the old Homœopathic College of

Penna. Later he established a sanitarium at Stamford, Conn., for the treatment of nervous and mental complaints. Upon the death of his wife, a few years ago, he gave up the sanitarium, and removed to the West. Dr. Foote was one of the organizers of the I. H. A., and always a firm believer in Hahnemannian Homœopathy. In 1884, Dr. Foote was chosen President of the I. H. A.; he always expressed great interest in its welfare, and entertained the most hopeful views of its success. A few years ago the Doctor became interested in investigating the so-called "Faith-cure," and recently published a brochure upon the subject.

---

### G. FELIX MATTHES.

Dr. Gustavus Felix Matthes, who died in New Bedford, March 17th, 1889, was one of the oldest physicians in the city. Death resulted from paralysis, produced by a fall which he received about two years ago. For the past year he had been confined to the house. Dr. Matthes was born in Schwedt, Prussia, on the last day of 1809. Choosing his father's calling, he entered the united Universities of Halle and Wittenberg, from which he received his medical degree in March, 1836, but he continued his studies for two years longer in Vienna, Prague, and Berlin. He commenced practice in the latter place in 1838, but in 1840 he removed to his native place, Schwedt.

Notwithstanding he had bitter prejudices against Homœopathy, yet, in 1845, his attention was drawn to it by the favorable results obtained by the neighboring owners of the large and costly herds of merinos, who had entirely discarded their professional veterinary surgeons, and adopted the homœopathic domestic practice. He soon became a thorough adherent of the despised system.

In 1849 he came to America, arriving in New York in July. His stay there was short, for in the following autumn he removed to Boston, where he practiced medicine for about a year. Then he located in New Bedford, where he has since resided.

He was a true homœopath, kind and genial, ever ready to assist the poor, and in scores of households, where he was a trusted adviser for many years, he was regarded with an affectionate appreciation such as few physicians can command. Dr. Matthes was always devoted to the interests of his patients, and thoroughly realized the responsibilities of his profession; no man was more highly esteemed by all, both as a physician and as a man.

